# Pediatric Paroxysmal Exercise-Induced Neurological Symptoms: Clinical Spectrum and Diagnostic Algorithm

**DOI:** 10.3389/fneur.2021.658178

**Published:** 2021-06-01

**Authors:** Federica Rachele Danti, Federica Invernizzi, Isabella Moroni, Barbara Garavaglia, Nardo Nardocci, Giovanna Zorzi

**Affiliations:** ^1^Unit of Child Neurology, Department of Pediatric Neuroscience, Fondazione Istituto di Ricovero e Cura a Carattere Scientifico (IRCCS) Istituto Neurologico Carlo Besta, Milan, Italy; ^2^Unit of Medical Genetics and Neurogenetics, Fondazione Istituto di Ricovero e Cura a Carattere Scientifico (IRCCS) Istituto Neurologico C. Besta, Milan, Italy

**Keywords:** paroxysmal dyskinesia, episodic ataxia, exercise intolerance, exercise, pediatric

## Abstract

Paroxysmal exercise-induced neurological symptoms (PENS) encompass a wide spectrum of clinical phenomena commonly presenting during childhood and characteristically elicited by physical exercise. Interestingly, few shared pathogenetic mechanisms have been identified beyond the well-known entity of paroxysmal exercise-induced dyskinesia, PENS could be part of more complex phenotypes including neuromuscular, neurodegenerative, and neurometabolic disease, epilepsies, and psychogenetic disorders. The wide and partially overlapping phenotypes and the genetic heterogeneity make the differential diagnosis frequently difficult and delayed; however, since some of these disorders may be treatable, a prompt diagnosis is mandatory. Therefore, an accurate characterization of these symptoms is pivotal for orienting more targeted biochemical, radiological, neurophysiological, and genetic investigations and finally treatment. In this article, we review the clinical, genetic, pathophysiologic, and therapeutic landscape of paroxysmal exercise induced neurological symptoms, focusing on phenomenology and differential diagnosis.

## Introduction

**Figure 1:**
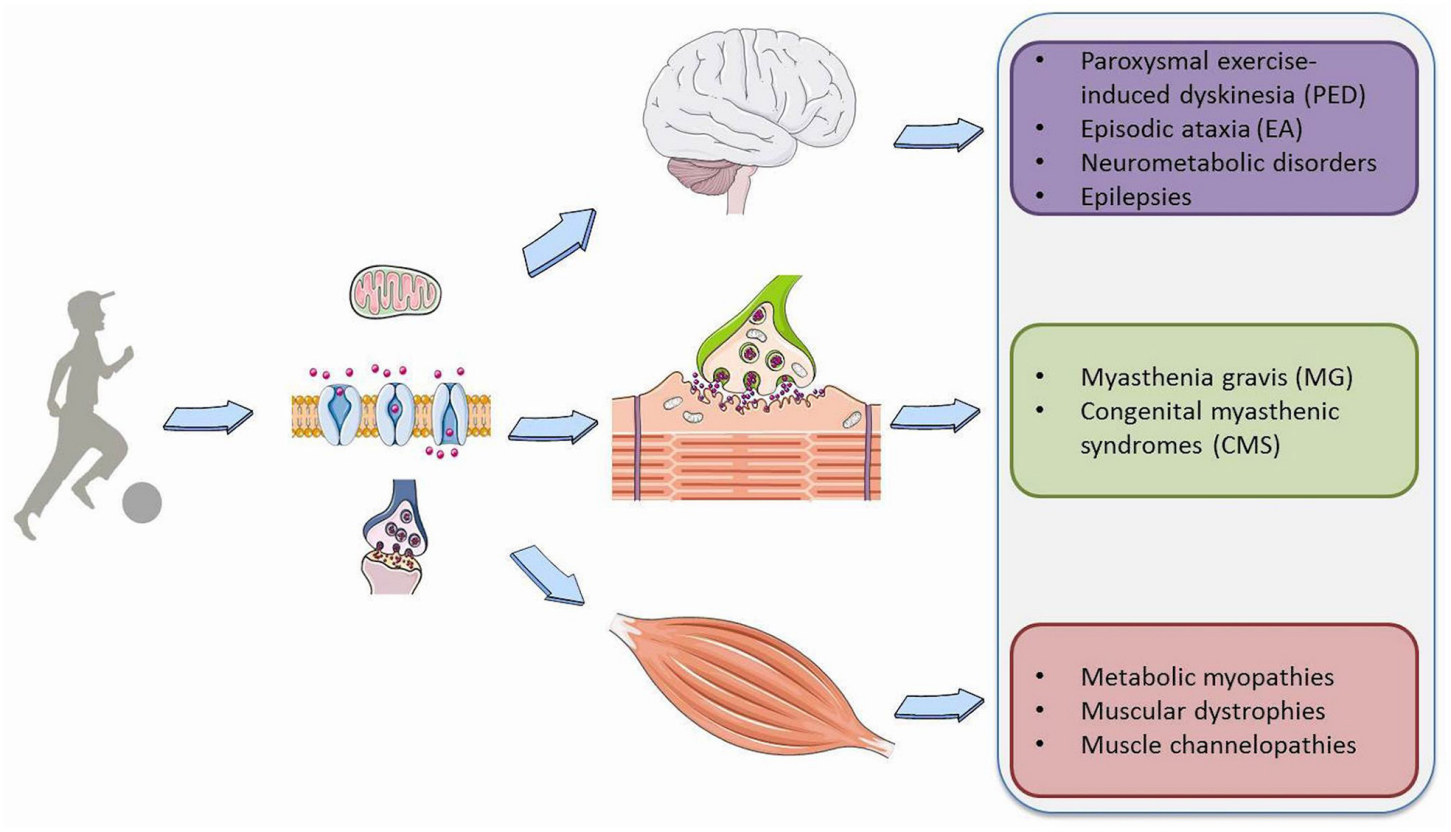
Overview of Paroxysmal exercise-induced neurological symptoms.

Physical exercise requires a profound adaptation of the whole body to be able to provide the increased energy needs essential for muscle contraction and the physiological functions of vital organs such as the heart, lungs and most importantly the brain. During exercise, based on its intensity and duration, a variety of metabolic pathways are simultaneously activated to produce ATP from different substrates, such as phosphocreatine, carbohydrates, free fatty acids, and branched-chain amino acids (1). Moreover, exercise results in a number of modifications in the synthesis and metabolism of brain neurotransmitters, action potential properties or synaptic transmission that are fundamental for signal transduction both in muscle and neuronal cells (2). Consequently, genetic or acquired defects disturb membrane excitability, synaptic transduction and transport, and storage, mobilization or utilization of the substrates involved in cell metabolism in the brain, muscles or neuromuscular junction (NMJ) (Figure 1). This leads to a wide spectrum of pediatric neurological symptoms characteristically elicited by prolonged physical exertion.

We propose to group these phenomena, which are frequently encountered in clinical practice, in a new clinical entity defined as Paroxysmal Exercise-induced Neurological Symptoms (PENS). They range from dyskinesia and ataxia to myalgia, cramping and rhabdomyolysis, myotonia, stiffness or weakness. They could be isolated or part of the phenotype of a number of neuromuscular, neurodegenerative, neurometabolic, epileptic and psychogenetic disorders. The wide number of genes involved in their pathogenesis reflects the high complexity of the causative molecular mechanisms (Table 1). The partial overlap and similarity among reported symptoms and the broad associated genetic heterogeneity make the differential diagnosis difficult and frequently delayed; however, a prompt diagnosis is required since some of these disorders may be treatable. Therefore, an accurate characterization of these symptoms is pivotal for orienting the extensive differential diagnosis through more targeted biochemical, radiological, and neurophysiological investigations and definitive genetic testing.

**Table 1:** Main genetic causes of PENS and related clinical, biochemical/histological/neuroimaging findings and treatments.

**Gene**	**Gene/Locus MIM number**	**Inheritance**	**Disease**	**Paroxysmal clinical features**	**Triggers**	**Other clinical features**	**Biochemical/histological findings**	**Neuroimaging**	**Specific treatment**
**Movement disorders**									
*SLC2A1*	138140	AD	Glucose transporter 1 deficiency	PED, EA, migraine, hemiplegic attack	Exercise, fasting, fever	Microcephaly, hypotonia, spasticity, seizures, DD/ID, dystonia, ataxia	↓CSF glucose, ↓CSF lactate, anemia		Ketogenic diet, triheptanoin
*TBC1D24*	613577	AR	Epilepsy, rolandic, with proxysmal exercise-induce dystonia and writer's cramp	PED	Exercise	Sizures, DD/ID, myoclonus, ataxia, extraneurological abnormalities		Normal, cerebral and/or cerebellar atrophy	AEDs
*ADCY5*	600293	AD	Benign hereditary chorea; dyskinesias	PED, PKD, PNKD, Nocturnal PxD	Exercise	DD/ ID, Axial hypotonia, Orofacial myoclonus, nonparoxysmal dystonia and chorea			AEDs, DBS?
*GCH*	600225	AD	DYT5	PED, painful stiffness or muscular cramps	Exercise	Dystonia and parkinsonism, marked diurnal fluctuation	↓ BH4, HVA and 5-HIAA		L-DOPA
*PARKIN*	602544	AR	PARK2 (Parkinson disease, juvenile, type 2)	PED	Exercise	Parkinsonism		Abnormal DaTSCAN	L-DOPA
*KCNA1*	176260	AD	EA1	EA	Eexercise, physical or emotional stress	Brief episodes of EA, interictal myokymia, progressive ataxia, epilepsy		Normal or cerebellar atrophy	CBZ, PHT, ACZM
*CACNA1A*	601011	AD	EA2	EA, Paroxysmal tonic upgaze, paroxysmal torticollis	Eexercise, physical or emotional stress	Prolonged episodes of EA, dystonia, DD/ID, epilepsy, progressive ataxia		Normal or cerebellar atrophy	ACZM, 4-APD, LEV
*PDHA1/ PDHX/ DLAT* (PDH complex)	300502/ 608769/ 608770	AR	Pyruvate dehydrogenase complex deficiency	PED, PNKD, EA	Exercise	DD/ID, epilepsy, progressive dystonia	↑blood and CSF lactate and pyruvate, ↑blood alanine, ammonia	BG T2W hyperintensities, BA, agenesis of corpus callosum, ↑lactate on MRS	Thiamine, ketogenic diet
*ECHS1*	602292	AR	Mitochondrial short-chain enoyl-CoA hydratase 1 deficiency	PED	Exercise	Leigh syndrome	↑ S-(2-carboxypropyl) cysteine and N-acetyl-S-(2-carboxypropyl) cysteine and urinary organic acids	Pallidal hyperintensities to Leigh-like abnormalities	Valine-restricted diet? detoxifying drugs?
*HIBCH*	610690	AR	3-hydroxyisobutryl-CoA hydrolase deficiency	PED	Exercise	DD/ID, Seizures, progressive dystonia	↑CK, ammonia, lactate; respiratory chain complex V deficiency in leukocytes	Hyperintensities to Leigh-like abnormalities	Valine-restricted diet? detoxifying drugs?
*ALDH5A1*	610045	AR	Succinic semialdehyde dehydrogenase (SSADH) deficiency	PED	Exercise	DD/ID, epilepsy	↑ GHB in biological fluids	Bilateral T2-weighted hyperintensity of pallidum and dentatum.	VGB
*DARS2*	610956	AR	Leukoencephalopathy with brain stem and spinal cord involvement and lactate elevation	Paroxysmal gait ataxia and areflexia	Exercise	Mild distal decrease in position and vibration sense, mild leg spasticity and hyperreflexia, progressive ataxia	↑blood and CSF lactate	Leukoencephalopathy with brain stem and spinal cord involvement, ↑lactate on MRS	ACZM
**Neuromuscular disorders**									
*PYGM*	608455	AR	McArdle disease (GSD V)	Pain on exercise, early fatigue, cramps, contractures	Exercise (within minutes)	Rhabdomyolysis, myoglobinuria, renal failure, “Second wind” phenomenon; abnormal ischemic forearm test; carbohydrate intake emeliorates exercise intolerance	CK chronically raised, ↑urate, and ammonia; myophosphorylase deficiency by histochemistry/biochemistry. Biopsy: minimal glycogen storage; many necrotic and regenerating fibers		Oral sucrose prior to exercise
*PFKM*	610681	AR	Tarui disease (GSD VII)	Muscle pain/ cramps, exercise intolerance, fatigue	Exercise (within minutes), illness; carbohydrate intake	Rhabdomyolysis, renal failure, carbohydrate intake exacerbates exercise intolerance ('out-of-wind' phenomenon)	↑CK, Haemolytic anemia, Hyperuricemia, bilirubin, reticulocitosis; PFK deficiency in muscle. Biopsy: muscle polyglucan and glycogen deposition		High protein diet, aerobic conditioning
*CPT2*	608836	AR	Carnitine palmitoyltransferase type II deficiency	Exercise intolerance, myalgia	Prolonged exercise/ heat or cold/ infection/ fasting/stress/general anesthesia	Deleyed Rhabdomyolysis, renal failure	CK normal between episodes, acute ↑CK after triggers; Abnormal acylcarnitine profile (increased C16+ C18:1)/C2 ratio); fibroblast CPT2 assay; muscle biopsy: lipid myopathy		Restriction of lipid intake, increased carbohydrate intake, Medium chain triglycerides (MCT oils), avoidance of fasting
*ACADVL*	201475	AR	Very long-chain acyl-CoA dehydrogenase deficiency	Exercise intolerance, myalgia	Prolonged exercise/heat or cold/infection/fasting/stress	Rhabdomyolysis, renal failure	CK normal between episodes, acute ↑CK after triggers, +/- hypoglycemia; increased serum long-chain acylcarnitines (↑C12, ↑↑C14:1, C14, C16, C16:1, C18:1); normal/low free carnitine; fibroblast FAO studies/enzyme assay; muscle histology:lipid myopathy		Avoidance of fasting, very low-fat, high-carbohydrate diet, with frequent feeding.
*HADHA/ HADHB*	609015	AR	Mitochondrial trifunctional protein deficiency	Muscle weakness, exercise intolerance	Exercise/Infection/heat or cold/fasting/stress	Rhabdomyolysis, renal failure, ±pigmentary retinopathy, progressive axonal sensory motor peripheral neuropathy, ataxic gait	CK normal between episodes, acute ↑CK after triggers; Increased serum long-chain 3-hydroxy- acylcarnitines (↑C14(OH), C16 (OH), C16:1(OH), C18(OH), C18:1(OH)); ddicarboxylic aciduria, fibroblast FAO studies/ enzyme studies (b-oxidation defect)		Low fat diet with restriction of long chain fatty acid intake and substitution with medium chain fatty acids. Avoidance of fasting and exposure to environmental extremes. Exercise limitation.
*RYR1*	180901	AD	Ryanodine receptor rhabdomyolysis-myalgia syndrome.	Rhabdomyolysis and/or exertional myalgia, exertional heat stroke	Exercise	Muscle hypertrophy, renail failure, malignant hyperthermia (MH) susceptibility	↑CK		Physical therapy, Dantrolene, beta-agonist (salbutamolo, albuterolo)
*CHRNE*	100725	AR	Congenital myastenic syndrome	Fatigable weakness	Exercise	Ptosis, ophthalmoparesis, facial, bulbar or generalized muscle weakness, spinal deformities and reduced muscle bulk			Acetylcholinesterase inhibitors (AChEIs) (e.g., pyridostigmine), 3,4-diaminopyridine (3,4-DAP), albuterol, ephedrine, fluoxetine, prednisone.
*CLCN1*	118425	AD/AR	Myotonia congenita: Thomsen's (AD) and Backer's (AR)	Myotonia	Exercise	Cold temeratures exacerbate symptoms, warm weather and alcohol are alleviating factors			Anticonvulsants (e.g., PHT, CBZ), anti-arrhythmic drugs (e.g., mexiletine, tocainide, flecainide, propafenone)
*SCN4A*	603967	AD	Paramyotonia congenita (PMC), sodium channel myotonia (SCM), HyperKPP and HypoKPP	Myotonia, paramyotonia, PP	Exercise	Myalgia, exercise intolerance	Abnormal potassium level		Correction of the potassium abnormality; Carbonic anhydrase inhibitors (ACZM, dichlorphenamide), Anticonvulsants (e.g., phenytoin, CBZ), anti-arrhythmic drugs (e.g., mexiletine, tocainide, flecainide, propafenone)
*CACN1AS*	114208	AD	HypoKPP	PP	Exercise, carbohydrate-rich meals		Anormal potassium level		Correction of the potassium abnormality; Carbonic anhydrase inhibitors (ACZM, dichlorphenamide)

*4-APD, 4-amynopiridine; ACZM, Acetazolamide; AR, Autosomic recessive; AD, autosomic dominant; AEDs, Anti-epileptic drugs; BG, Basal Ganglia; BH4, Tetrahydrobiopterin; CBZ, carbamazepine; CK, Creatine Kinase; CSF, Cerebro Spinal Fluid; DBS, Deep Brain Stimulation; DD, Developmental delay; EA, episodic ataxia; FAO, fatty acid oxidation; GHB, 4-hydroxybutyric acid; GSD, glycogen storage disease; HIAA, 5-Hydroxyindoleacetic Acid; HVA, Homovanillic Acid; ID, Intellectual disability; LEV, Levetiracetam; MRS, Magnetic resonance spectroscopy; PED, Paroxysmal exercise-induced dyskinesia; PHT, Phenytoin; PKD, Paroxysmal kynesigenic dyskinesia; PNKD, Paroxysmal non-kynesigenic dyskinesia; PP, Periodic Paralysis; PxD, Paroxysmal dyskinesias; T2W, T2-weighted; VGB, Vigabatrin*.

This review examines the clinical, genetic, pathophysiologic and therapeutic landscape of PENS, classifying them based on the type of the main paroxysmal symptom and focusing on pathophysiology and differential diagnosis.

## Pediatric Paroxysmal Exercise-Induced Neurological Symptoms: Phenotypic and Genotypic Spectrum

### Movement Disorders

#### Paroxysmal Exercise Induced Dyskinesia (PED)

Paroxysmal Exercise Induced Dyskinesias (PED) are genetically heterogeneous conditions characterized by recurrent episodes of sudden, involuntary movement that are typically induced by prolonged exercise (3). They commonly present during infancy or childhood with dystonic or choreic attacks, isolated or in combination, that usually affects the part of the body that is involved in the exercise, such as leg or arm dystonia after prolonged walking or writing. The duration and frequency of PED are very variable, but the attacks usually last 5–30 min and recur daily or a few times per month. PED can be the only or prominent manifestation of a disease or can be associated with other paroxysmal manifestations (epilepsy, migraine) and\or interictal neurological abnormalities. Although rare, they should be promptly recognized since some of them are treatable (4).

**Figure 2:**
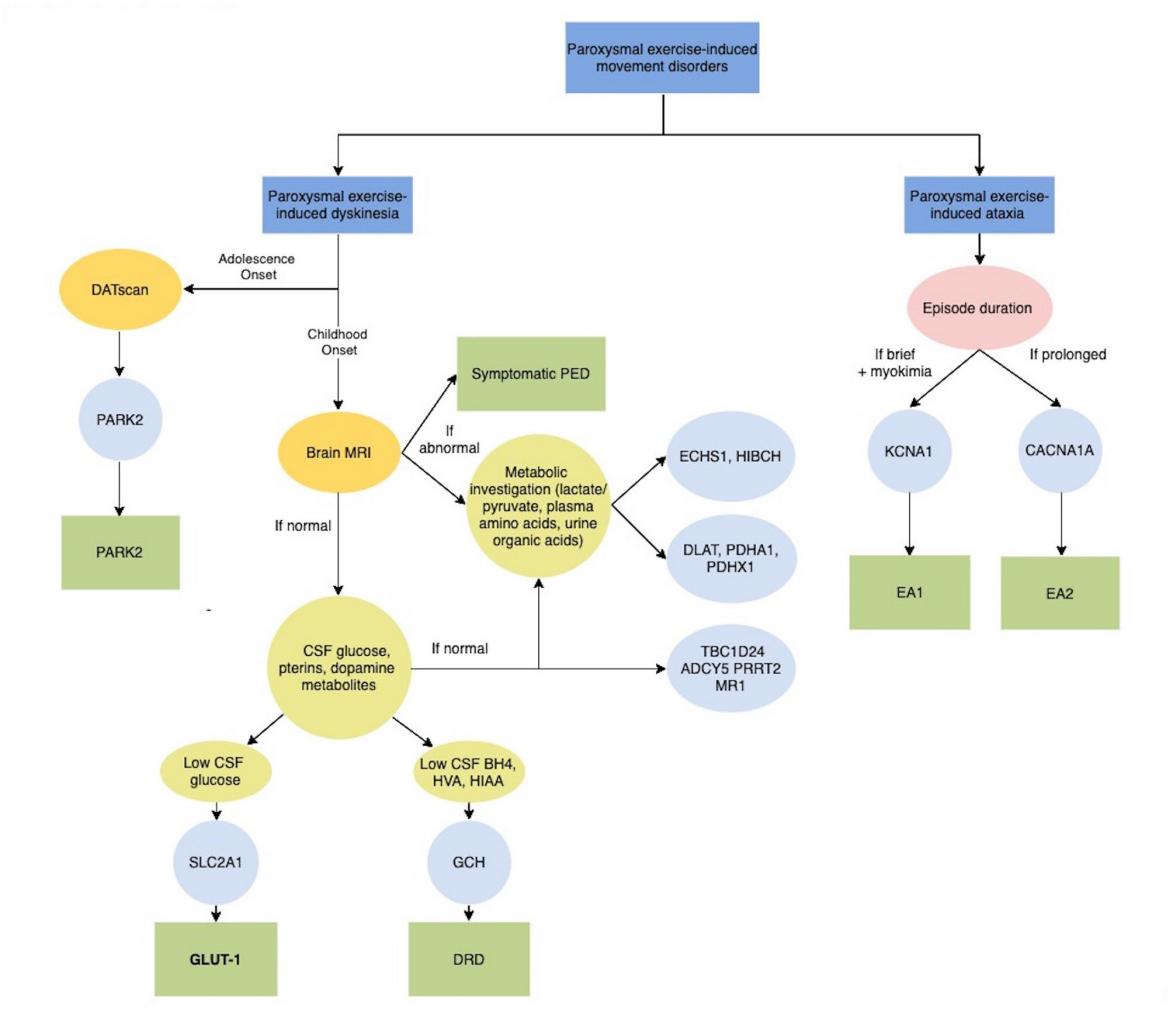
Diagnostic algorithm for Paroxysmal exercise-induced movement disorders. ADCY5, Adenylate Cyclase 5; BH4, tetrahydrobiobterin; CACNA1A, Calcium Channel, Voltage-Dependent, P/Q Type, Alpha-1a Subunit; CSF, Cerebrospinal fluid; DATscan, Dopamine transporter (DAT) single photon emission computerized tomography (SPECT) imaging technique; DLAT, Dihydrolipoamide acetyltransferase; DRD, Dopa-Responsive Dystonia; EA1, Episodic Ataxia, Type 1; EA2, Episodic Ataxia, Type 2; ECHS1, Short-chain enoyl-CoA hydratase; GLUT-1, Glucose Transport Defect, Blood-Brain Barrier, Deficiency Syndrome 1; GCH, GTP Cyclohydrolase I; HIAA, 5-hydroxyindolacetic acid; HIBCH, 3-Hydroxyisobutyryl-CoA hydrolase; HVA, Homovanalinaic Acid; KCNA1, Potassium Channel, Voltage-Gated, Member 1; MRI, Magnetic Resonance Imaging; MR-1, Myofibrillogenesis Regulator 1; PARK2, Parkinson disease-2; PDHA1, Pyruvate dehydrogenase E1-alpha deficiency; PDHX1, Pyruvate Dehydrogenase Complex Component X; PED, Paroxysmal exercise-induced dyskinesia; PRRT2, Proline-Rich Transmembrane Protein 2; SLC2A1, Solute Carrier Family 2 (Facilitated Glucose Transporter), Member 1; TBC1D24, Tbc1 Domain Family, Member 24.

Heterozygous mutations in the *SLC2A1* (solute carrier family two, member one) gene encoding for the glucose transporter gene 1 (GLUT1) are the main causative defect of PED (5, 6). These mutations can be isolated or in the context of a more complex phenotype (GLUT1 deficiency syndrome, GLUT1-DS), which include epilepsy, ataxia, spasticity, dystonia, intellectual disability and other paroxysmal events such as migraine, hemiplegic attacks or episodic ataxia (EA) (7). Typically, the onset is during childhood in otherwise normal subjects or those with a mild interictal neurological abnormality or a history of epilepsy with generalized seizures. Attacks are predominantly dystonic or choreo-dystonic with a focal or unilateral distribution and generalization is rare (5–9). Besides exercise, fasting or fever are also reported as triggering factors due to energy metabolism defect (3, 7). Glucose is the essential substrate for brain energy metabolism, especially in infants and children where the brain can consume as much as 80% of the body's total glucose supply in the resting state (9). GLUT1 is constitutively expressed in most tissues but predominantly expressed in erythrocytes, brain endothelial cells, and astrocytes and is fundamental for the passage of glucose across the blood–brain barrier and cell membranes. Consequently, a low concentration of glucose in cerebrospinal fluid (CSF) and a decreased CSF to blood glucose ratio are the best biochemical clues to diagnosis, although they are not constantly found. Molecular analysis of the *SLC2A1* gene confirms the diagnosis (Figure 2). The mutation type and resulting residual GLUT1 activity are responsible for the heterogeneity and severity of the phenotype (10). Exon 4, which encodes the fourth transmembrane domain of GLUT1, has been suggested as a vulnerable region of the protein, and mutational hotspots have also been reported (11). The ketogenic diet is the gold-standard therapy for epilepsy and paroxysmal and non-paroxysmal movement disorder (12), however, the dietary constraints and side effects may be poorly tolerated and inefficacy has also been reported (13). Treatment with triheptanoin has resulted in a dramatic and sustained reduction of non-epileptic paroxysmal events in a group of GLUT1-DS patients who were not on the ketogenic diet (13).

Bi-allelic mutations in the *TBC1D24* gene, which encodes for a presynaptic protein involved in vesicle tracking, have been described in a wide range of neurological disorders that include epileptic syndromes, DOORS syndrome (deafness, onychodystrophy, osteodystrophy, intellectual disability and seizures) or syndromic and non-syndromic deafness (14). Recently PED as a prominent clinical feature associated with epilepsy has been reported in few patients (15–17). Episodes starts usually between 1 and 2 years of age and are characterized by dystonia triggered by exercise or fatigue; patients present with episodes of trunk bending laterally or backward without clear discomfort, some other cases have arm dystonia after writing or performing motor tasks. The phenomenology of the movement disorder has also be reported as hyperkinetic (tremor or fine myoclonus), involving the oromandibular region or the voice after prolonged chewing or singing (17). Episodes last seconds to hours and are alleviated or ceased by resting. No specific treatment results in marked benefit, although a possible effect of acetazolamide (17) and carbamazepine (16) has been suggested. Therefore, avoidance of precipitating factors remains the most effective therapeutic strategy. For cases with long follow-up, a reduction of the severity of the paroxysms although persisting into adulthood has been documented (15).

Mutations in *ADCY5*, which encodes for adenylate cyclase type 5 (AC5), a membrane-bound enzyme highly expressed in striatal neurons, were first described in a large dominant kindred with affected cases presenting with an early-onset hyperkinetic movement disorder defined Familial Dyskinesia with Facial Myokymia (18). The spectrum of ADCY5- related movement disorder have been gradually expanded to include NKX2-1 negative patients with benign hereditary chorea (19), childhood-onset dystonia, chorea and myoclonus and patients diagnosed with dyskinetic cerebral palsy. The hallmarks of the disease are episodic exacerbations of baseline movement during sleep and when awake (20, 21). Delayed motor and/or language milestones with or without axial hypotonia are frequently reported. The paroxysmal dyskinesias are mostly generalized, with rapid dystonic and ballistic movements, and can last for hours and are sometimes painful. Different triggers, even in the same patient, can provoke the paroxysms, including, but not exclusively, physical exercise (21). Although patients have a chronic, sometimes slowly progressive, movement disorder, they usually complain the paroxysmal dyskinesias being the most disabling symptoms. The antiepileptic drug, acetazolamide, benzodiazepines, tetrabenazine, propranolol have been tested with little or no benefit; recently pallidal deep brain stimulation improved both the chronic and paroxysmal movement disorder in few patients (22). The disease is transmitted with an autosomal dominant mode of inheritance and the majority of reported *ADCY5* mutations are located in exons 2 and 10, at residue 418 and 1029, respectively (20, 21).

Moreover, exercise is reported to be a fairly frequent trigger in patients carrying *PRRT2* and *MR-1* mutations (3), which are more typically responsible for paroxysmal kinesigenic dyskinesias (PKD) and paroxysmal non-kinesigenic dyskinesias (PNKD) (Figure 2) (11).

In rare cases, PED can occur as the first symptom of juvenile parkinsonism and DOPA-responsive dystonia (DRD) due to *PARK2* and *GCH1* mutations respectively, supporting the role of neurotransmitter deficiency, together with brain energy deficiency, in the pathophysiology of PED (Figure 2).

The E3 ubiquitin ligase PARKIN (encoded by *PARK2*) works together with PINK1 (a mitochondrial-targeted kinase encoded by *PARK6*) in a common pathway to remove dysfunctional mitochondria by autophagy. Therefore *PARK2* and *PARK6* mutations result in the accumulation of dysfunctional mitochondria that release reactive oxygen species (23). The mean onset of juvenile parkinsonism is around age 30 years, but adolescent cases are recognized and PED, typically presenting with leg or foot dystonic posturing elicited by prolonged walking is particularly frequent in this age range (24, 25). *GCH1* codes for GTP cyclohydrolase I, a rate limiting enzyme in the synthesis of tetrahydrobiobterin (BH4) from GTP. Autosomal dominant GCH deficiency, commonly known as Dopa-responsive dystonia, Segawa disease, or DYT5, is defined by the association of dystonia, tremor or bradykinesia, marked diurnal fluctuation, specific CSF pattern (low levels of BH4, homovanillic acid, and 5-hydroxyindolacetic acid) and sustained response to L-dopa administration (Figure 2) (24, 26). PED is often the presenting symptom of the disease; notably, together with abnormal posturing, patients frequently complain of painful stiffness or muscular cramps, suggesting a differential diagnosis among neuromuscular disorders. As the disease progresses dystonia involves other body regions and parkinsonian signs also become evident, but there are reported patients in which PED remained the unique manifestation (27).

#### Paroxysmal Exercise Induced Ataxia

Episodic ataxia (EA) comprises a heterogeneous group of genetic conditions characterized by recurrent episodes of imbalance, incoordination and postural instability (28). EA can result from mutations in several genes that play important roles in the nervous system. Physical exercise has been reported to trigger episodes of ataxia due to mutations in *KCNA1* and *CACNA1A*, which are responsible for episodic ataxia types 1 and 2, respectively (Figure 2). These genes encode for proteins that are involved in the transport of ions across cell membranes and play important roles in excitatory neurotransmission (28). As for PED, exercise-induced episodes are usually associated with a variety of other paroxysmal manifestations and persistent neurological symptoms.

Episodic ataxia type 1 (EA1) is characterized by brief episodes of ataxia with myokimia and later, the development of cerebellar signs. Onset is usually before the age of 10 years. Ataxic symptoms such as dizziness, unsteady, wide-based gait, incoordination, and dysarthria are often accompanied by weakness, stiffness, headache, nausea, vomiting, and visual disturbance. Myokimia usually involves the periocular or perioral region or of the distal extremities. It can be sometimes be misinterpreted as tremor or chorea, making the differential diagnosis between PED and EA often difficult. Physical exertion, emotional stress and environmental temperature are the usual triggers for attacks. Episodes are usually brief, but the severity may range from inability to stand and walk to independent walking. The frequency of attacks is variable, daily to monthly, and tends to decrease during adulthood. Up to 20% of EA1 patients develop persistent cerebellar signs. Carbamazepine is the most useful drug in EA1, but some benefits have been reported with valproic acid and acetazolamide (28, 29). EA1 is caused by heterozygous mutations in *KCNA1*, which encodes the a1 subunit of a neuronal voltage-gated potassium channel, Kv1.1, that is highly expressed in the interneurons and Purkinje cells in the cerebellum and plays an important role in the inhibition of the cerebellar outputs. Around 30 *KCNA1* variants have been identified so far and the majority of them are missense mutations (30).

Episodic ataxia 2 (EA2), the most common subtype of EA, is a well-characterized genetic condition in which patients experience prolonged episodes of incoordination from early childhood, vertigo, dysarthria and often general weakness (31). Similarly to EA1, common triggers are exercise, physical or emotional stress but paroxysms can also arise spontaneously. Episodes are highly disabling and can last several days. As the disease progresses, interictal ataxia and nystagmus appear. Many cases with atypical features such as late onset, or paroxysmal tonic upward gaze in infancy or the appearance of dystonia, epilepsy and cognitive impairment are reported. Acetazolamide is the drug of choice to prevent attacks, both in adults and children. In cases of unresponsiveness or side effects, 4-aminopyridine has been demonstrated to be effective in a controlled trial (32). EA2 is caused by heterozygous mutations in *CACNA1A*, which encodes the a1A subunit of a neuronal voltage-gated calcium channel, Cav2. This channel, widely expressed in the Purkinje and granule cells of the cerebellum, mediates calcium entry and firing of the cells (28).

#### Neurometabolic Disorders

PED has been recently associated with the deficiency of a number of mitochondrial enzymes involved in energy production and branched-chain amino acids (BCAA; leucine, isoleucine, and valine) catabolism. They include Pyruvate dehydrogenase (PDH) complex, short-chain enoyl-CoA hydratase (ECSH1) and 3-Hydroxyisobutyryl-CoA hydrolase (HIBCH) (Figure 2) (33, 34).

PDH complex catalyses the oxidative decarboxylation of pyruvate with the production of acetyl-CoA; therefore, it connects the glycolytic pathway to the Krebs cycle and plays a central role in glucose metabolism in fed and fasting states. PDH complex is composed of three catalytic subunits: pyruvate dehydrogenase (PDH; E1, a heterotetramer of 2 subunits encoded by *PDHA1* and *PDHB1* genes), dihydrolipoamide acetyltransferase (E2, encoded by *DLAT* gene), and dihydrolipoamide dehydrogenase (E3, encoded by *DLD* gene), and of an additional component, the E3-binding protein (encoded by *PDHX1*) (35, 36). *PDHA1, DLAT*, and *PDHX1* mutations have been linked to continuously expanding phenotypes inherited with X-linked (*PDHA1* mutations, representing the main cause of PDH deficiency) or autosomal recessive pattern (*DLAT* and *PDHX1* mutation). Clinical findings range from severe infantile lactic acidosis to milder chronic neurological disorders including intermittent and recurrent acute neurological symptoms such as episodic ataxia, peripheral weakness, and movement disorders such as PED and PNKD (36–38). In few patients recurrent dystonic or hemidystonic attacks have been described; they are triggered by prolonged walking and running and occur as a unique clinical manifestation or within complex neurological phenotypes (39–41). Elevated serum lactate and bilateral pallidal hyperintensities, typical hallmarks of the disease, could be absent, particularly at onset (39). PDH deficiency is a potentially treatable disorder, partially responding to ketogenic diet and thiamine supplementation (42).

ECHS1 and HIBCH enzymes play a major role in the valine catabolic pathway and fatty acids degradation. Valine is essential for regulating protein synthesis, neurotransmission, and energy production (43). Moreover, recessive mutations in those genes generate secondary deficiencies in the mitochondrial oxidative phosphorylation system and PDH complex. Interestingly, specific dietary regimens for reducing the workload on the valine catabolic pathway, associated with detoxifying drugs, such as cysteamine and N- acetylcysteine, for replenishing intramitochondrial glutathione are a potential treatment strategy for both ECSH1 and HIBCH defects (42).

Recessive mutations in *ECHS1* have been reported as a novel cause of Leigh syndrome or atypical forms with later onset (34). Interestingly recent reports widened the clinical range of ECHS1-related disorders including few normally developing unrelated children with paroxysmal and frequently asymmetric dystonic episodes in the lower limbs lasting few minutes; most of them harbored the c.518C>T (p.A173V) variant (44–47). Elevated S-(2-carboxypropyl)cysteine and N-acetyl-S-(2-carboxypropyl)cysteine and urinary excretion of organic acids (2,3-dihydroxy-2-methylbutyric acid, 3-MGA, 3-HIVA, and EMA) and T2-weighted bilateral abnormalities at the level of the globus pallidus have been suggested in diagnosis, even in the mildest form (34, 44).

Similarly, autosomal recessive *HIBCH* mutations have been reported as a rare cause of early-infantile mitochondrial encephalopathy with progressive dystonia. A milder phenotype was identified in a 6-year-old girl suffering from isolated exercise-induced dystonic posturing in both lower extremities and the waist associated with bilateral symmetrical hyperintensities in the globus pallidus on brain MRI. Interictal neurological examination was unremarkable. Laboratory findings showed elevated serum creatine kinase (CK), ammonia, lactate and respiratory chain complex V deficiency in blood peripheral leukocytes. The patient demonstrated marked clinical and neuroradiological improvements after treatment with a low-valine diet (48). An increase of 3-hydroxy-isobutyryl-carnitine has been reported as a distinguishing finding of HIBCH from ECSH1 deficiency (33, 34).

PED has been described in two Italian siblings with mutations in *ALDH5A1* encoding for another mitochondrial enzyme, Succinic semialdehyde dehydrogenase (SSADH) that results in aberrant metabolism of the neurotransmitter GABA, the main inhibitory neurotransmitter of the central nervous system (49). They presented with early onset developmental delay and ID associated with dystonic postures of upper limbs and seizures. During adolescence, they exhibited PED that responds to vigabatrin which irreversibly inhibits GABA transaminase, and brain MRI showed bilateral T2-weighted hyperintensity of pallidum and dentatum. This rare autosomal recessive disorder exhibits a highly phenotypic heterogeneity, encompassing varying degrees of developmental delay, hypotonia, ataxia, seizures and movement disorder such as dystonia or choreoatetosis. Elevations of 4-hydroxybutyric acid (GHB) in biological fluids could guide the diagnosis. Treatment with vigabatrin resulted in mixed outcomes (50, 51).

Juvenile onset exercise-induced paroxysmal gait ataxia and areflexia of the upper and lower extremities are reported in a single case an atypical phenotype associated with a novel homozygous mutation c.1825C>T (p.R609W) in the *DARS2* gene. Heterozygous mutations in this gene, encoding a mitochondrial aspartyl-tRNA synthetase, are typically responsible for a more severe presentation defined as leukoencephalopathy with brain stem and spinal cord involvement and brain lactate elevation (LBSL) (52). The patient disclosed a brain MRI compatible with several major and supportive MRI criteria for LBSL and lactate peak in spectroscopy but permanent cerebellar ataxia or gait spasticity, hallmarks for this disease, were absent (53). Acetazolamide was effective in achieving good control of attacks (52).

### Neuromuscular Disorders

Exercise may induce a wide range of symptoms that point to a primary impairment of muscles or NMJ. These symptoms can be clustered into two distinct clinical categories, named Exercise intolerance and Paroxysmal exercise induced stiffness or weakness.

**Figure 3:**
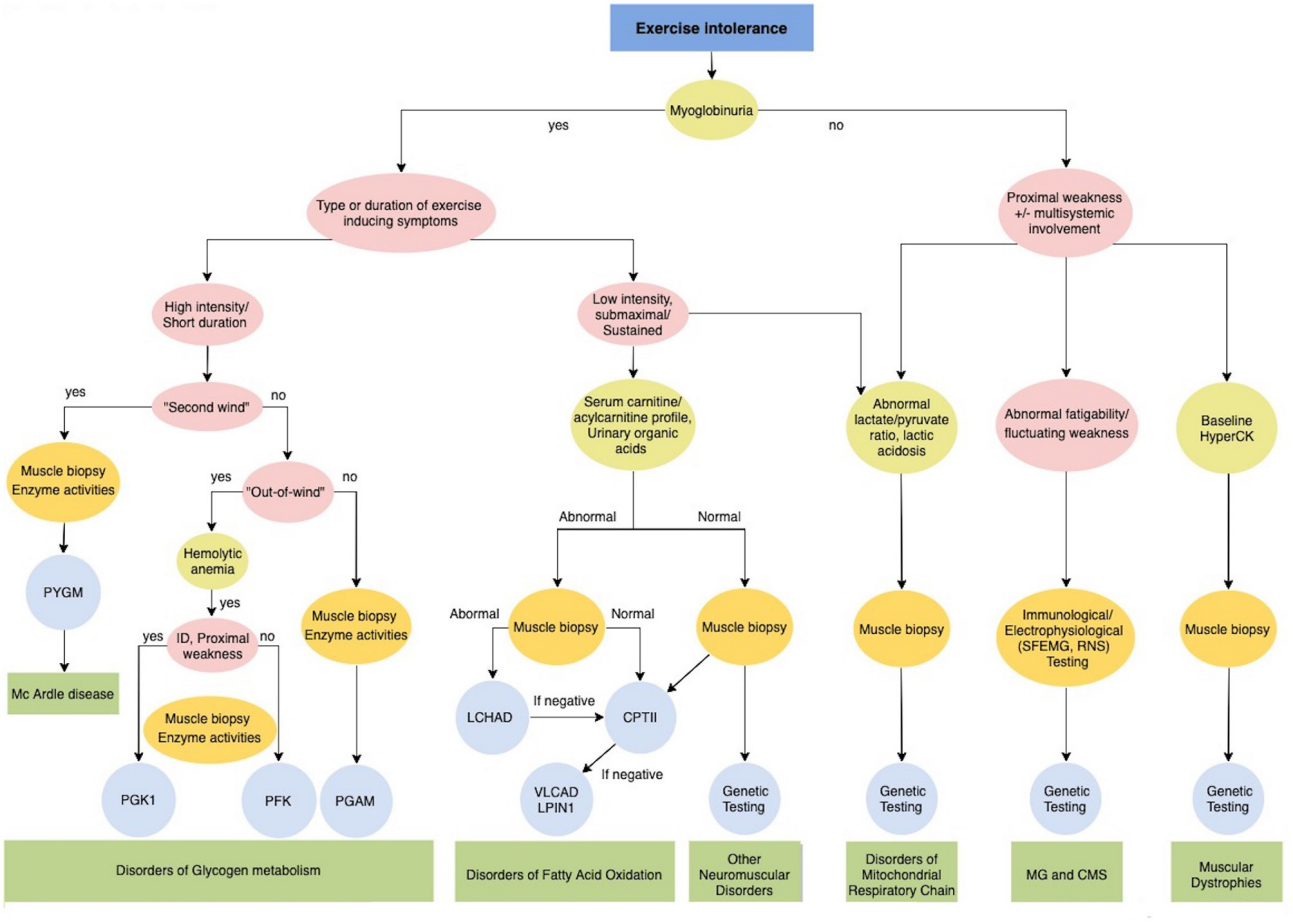
Diagnostic algorithm for Exercise intolerance. CMS, Congenital myasthenic syndromes; CPTII, carnitine palmitoyltransferase II; ID, intellectual disability; LCHAD Long-chain 3-hydroxyacyl-CoA dehydrogenase; LPIN1, Phosphatidate Phosphatase; MG, Myasthenia gravis; PFKM, phosphofructokinase; PGAM, Phosphoglycerate mutase; PGK1, Phosphoglycerate kinase 1; PYGM, phosphorylase; VLCAD, very long-chain long-chain acyl-CoA dehydrogenase.

**Figure 4:**
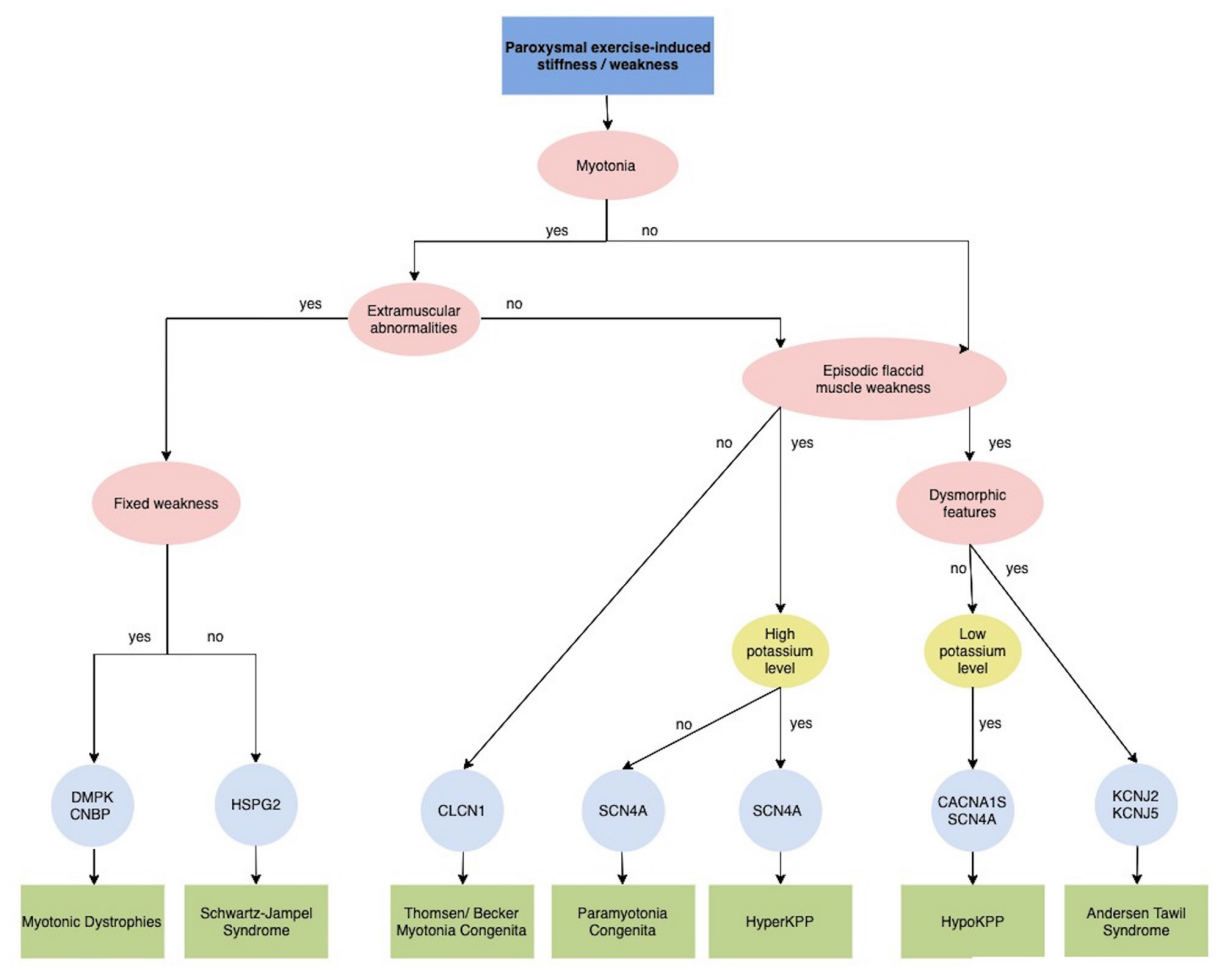
Diagnostic algorithm for Paroxysmal exercise-induced stiffness/weakness. CACNA1S, calcium voltage-gated channel subunit alpha1 S; CLCN1, chloride channel 1; CNBP, CCHC-type zinc finger, nucleic acid binding protein; DMPK, dystrophia myotonica protein kinase; HSPG2, heparan sulfate proteoglycan of the basement membrane; HyperPP, Hyperkalemic periodic paralysis; HypoPP, Hypokalemic periodic paralysis; KCNJ2, potassium voltage-gated channel subfamily J member 2; KCNJ5, potassium voltage-gated channel subfamily J member 5; SCN4A, sodium voltage-gated channel alpha subunit 4.

Exercise intolerance includes a variable combination of fatigue, muscle cramps and myalgia that causes inability or a decreased ability to perform physical exercise at the normally expected level based on age and sex. It manifests in metabolic myopathies as a consequence of a failure to meet the energy requirements of a motor task, and in addition in Myasthenia Gravis and Congenital Myasthenic Syndromes (Figure 3). Furthermore, exercise could induce paroxysmal weakness or stiffness in patients with Myotonic Dystrophies and Primary Muscle Channelopathies that include Non-dystrophic Myotonias and Periodic Paralysis (Figure 4).

Detailed clinical history must include previous symptoms, anatomical distribution and sequence progression pattern, associated features and family history. In particular, a history of dark colored urine or myoglobinuria should be acquired. Myoglobinuria as a sign of rhabdomyolysis strongly suggests a metabolic myopathy (Figure 3). Specific investigation of the intensity and duration of exercise and factors which worsen or improve symptoms are fundamental for orienting etiological definition. Neurological and general examination should assess dynamic and static symptoms and multisystemic involvement.

An evaluation must involve blood testing (CK, acylcarnitine profile, lactate/pyruvate, amino acids), urine organic acids, exercise testing, neurophysiological, muscle biopsy for histology, ultrastructure, enzyme testing, eventually brain MRI/spectroscopy, and targeted or untargeted genetic testing (54, 55). If all studies are normal, a psychogenic cause should be considered (56).

#### Metabolic Myopathies

Metabolic myopathies are a heterogeneous group of rare genetic disorders characterized by muscle dysfunction secondary to an acute bioenergetic crisis. This is due to defects in the biochemical pathways of storage, mobilization and utilization of the substrates involved in muscle cell metabolism (57, 58).

Metabolic myopathies typically present during childhood with a wide clinical spectrum including acute forms of exercise intolerance with the normal interictal clinical examination; or progressive forms characterized by proximal muscle weakness frequently combined with multisystem involvement (59–61). Three main types of metabolic myopathies are distinguished according to the impaired metabolic pathway: (1) disorders of glycolytic and glycogenolytic metabolism; (2) disorders of fatty acid metabolism; and (3) disorders of the mitochondrial respiratory chain. Establishing the type of activity that produces the acute phenotype is essential for better orienting toward the impaired metabolic pathway (Figure 3) (58, 60).

More specifically, glycogen breakdown and glycolysis defects manifest with episodes of exercise intolerance, muscle pain, contractures and hyperCKemia early after (usually within 5 min) short intense, isometric activity such as running or lifting heavy objects. Basal CK level is usually elevated and examination is usually normal in children, while interictal weakness can emerge in adults. Distinguishing features can help narrow the diagnosis (60, 62). In particular, a marked improvement in the exercise tolerance about 10 min into aerobic, dynamic, large muscle mass exercise followed by the ability to continue exercise without difficulty (“second wind” phenomenon) directs diagnosis toward muscle phosphorylase deficiency or McArdle's disease (Glycogen storage disease, or GSD, type V), the most common disorder of carbohydrate metabolism (63). Non-ischemic forearm exercise testing is abnormal, showing only an increase in ammonia and constant levels of lactic acid and pyruvate. The absence of myophosphorylase and muscle biopsy and genetic mutation analysis confirmed the diagnosis; screening of the common mutations (p.R50X, p.W798R, p.G205S in Caucasians and p.Phe710del in Japanese) in *PYGM* should be evaluated (63).

Glucose or sucrose intake usually ameliorates symptoms in McArdle's disease, while exacerbating them in PFK deficiency (“out-of-wind” phenomenon of Tarui's disease or GSDVII), the second most common glycolytic disorder. Haemolytic anemia has been described in patients with *PFKM* but also in *PGK1* mutations; in the latter encephalopathy and hyperammonaemia are typically reported. Other less frequent muscle glycogenosis with acute exercise-induced phenotype are due to distal glycolytic defects- GSD IX, X and XI (*PHKA1, PGAM* and *LDHA* mutations respectively), characterized by abnormal forearm exercise test (reduced increased of lactate levels) (61). Skin rush during summer or uterine stiffness during pregnancy orient toward *LDHA* mutations; bifid uvula and hepatopathy are described in Phosphoglucomutases 1 (*PGM1*)-associated myopathy (GSD XIV); recessive mutations in the *ENO3* gene cause GSD XIII or muscle β-enolase deficiency; phenotype elicited by strenuous exertion and isometric muscle activity is usually milder than GSD V and “second wind” phenomenon is not observed (60–62, 64). Exercise intolerance could occur even in other GSD in the context of prominent progressive neuromuscular features such as Pompe's disease due to *GAA* mutation; however proximal weakness, fixed contractures, and cardio-respiratory involvement; GSD III and IV distal muscle weakness and liver involvement. If suggesting clinical key features are identified, single gene testing should be undertaken; otherwise, muscle biopsy for histochemical and enzymatic testing could direct diagnosis (60–62). Muscle biopsy is typically unremarkable in GSDV, while increased glycogen concentration by periodic acid-Schiff (PAS) staining and electronic microscopy can be disclosed in GSDII (65). Currently, there is no specific treatment for glycogen storage diseases that results in exercise-induced symptoms, apart from avoiding intense physical exercise and treating symptoms. A low dose of creatine monohydrate may provide a mild benefit in *PYGM* mutations. Immediate pre-exercise carbohydrate improves symptoms in the glycogenolytic defects (such as GSDV) but can exacerbate symptoms in glycolytic defects (such as GSDVII) (63).

Long chain fatty acids represent the main key energy source for muscle during prolonged, low intensity activity. Recurrent episodes of myalgia, fatigue, rhabdomyolysis precipitated by prolonged sub maximal aerobic exercise (or after other triggering events such as fasting, illness or emotional stress), should lead clinicians to consider defects of fatty acid metabolism (60, 62). The most common is Carnitine palmitoyltransferase II (CPTII) enzyme deficiency, which is due to recessive mutations in *CPT2* gene. This gene is part of the long chain fatty acids transportation system through the mitochondrial membrane and more than 30 pathogenic mutations have been identified, with p.S113L identified as the most common (66). Investigations usually disclose abnormal serum carnitine/acylcarnitine profile, with increased long-chain acylcarnitine. Ictal serum CK can be markedly elevated while the basal one is normal, as well as neurological examination. Very-long-chain acyl-CoA dehydrogenase (VLCAD) catalyses the initial step of mitochondrial beta-oxidation of long-chain fatty acids. Its deficiency is caused by recessive mutations in the *ACADVL* gene, which manifest in an infantile form complicated by severe cardiomyopathy or with an adult-onset phenotype clinically indistinguishable from adult CPTII deficiency. Plasma acylcarnitines pattern during periods of metabolic stress shows increased long chain intermediate, likewise to long-chain 3-hydroxyacylCoA dehydrogenase (LCHAD) deficiency due to mutation in the mitochondrial Trifunctional Protein (MTP). This is a very rare fatty acid oxidation (FAO) defect in which myoglobinuria is often associated with life-threatening respiratory distress and/or axonal sensory-motor neuropathy (62). Muscle biopsy is usually uninformative in CPTII and VLCAD, while in LCHAD a chronic denervation or rarely lipid storage could be disclosed (61). Therapeutic recommendations include the avoidance of precipitating factors, such as prolonged fasting, aerobic exercise, infections, exposure to cold and intensive saline, glucose solution administration, and monitoring of electrolytes, kidney and cardiac functions in the case of rhabdomyolysis.

Mitochondrial myopathies encompass a broad and heterogeneous group of rare genetic progressive conditions caused by mutations in genes in the nuclear DNA (nDNA) and mitochondrial DNA (mtDNA) that impair the oxidative phosphorylation (OXPHOS) in the mitochondria (67, 68). The consequence is a deficit in energy production in the form of adenosine triphosphate (ATP), with muscles being the most affected tissue. Mitochondrial myopathies rarely manifest with acute myalgia and myoglobinuria, such as in complex I, III (cytochrome b) and IV (cytochrome c oxidase or COX) (61, 68, 69). However, recurrent exercise intolerance and premature fatigue are frequently reported in mitochondrial myopathies. Comparable to fatty acid oxidation defects, symptoms usually manifest after prolonged submaximal leisurely activity or during fasting, intercurrent illnesses or fever. Exercise induced symptoms may manifest as isolated features or associated with progressive weakness and multisystemic involvement (65). Muscle weakness typically affects extraocular muscles (ptosis, ophthalmoparesis, or both), but frequently spreads to bulbar, limb or axial muscles. Identification of an accompanying multisystem disease, variably including cognitive impairment, encephalopathy, peripheral neuropathy, epilepsy, stroke-like events, cardiomyopathy, hepatopathy and nephropathy, gastrointestinal dysmotility, diabetes, or maternal inheritance pattern increases clinical suspicion of mitochondrial disease (65, 69). Exercise intolerance or rhabdomyolysis could be part of the classic maternally inherited mitochondrial encephalomyopathies such as MELAS, MERRF, MNGIE or Kearns-Sayre Syndrome/C-PEO (61) or secondary to a number of mitochondrial dysfunction, such as complex III (cytochrome b) and IV (cytochrome c oxidase or COX) such us tRNA mutations and Coenzyme Q10 deficiencies (61, 68). Notably, the latter form responds to CoQ10 supplementation (60). Laboratory investigations should include serum lactate and pyruvate (increased ratio) and CK (normal or mildly elevated), muscle biopsy (typically displaying ragged red fibers (RRF), COX-negative fibers), and measurement of the activities of respiratory chain complexes (61).

The deficiency of muscle-specific phosphatidic acid phosphatase (LPIN1) regulates lipid metabolism and the mitochondrial respiratory chain. It is recognized as a common cause of AR recurrent and life-threatening acute rhabdomyolysis of childhood-onset, sometimes triggered by exercise but mainly by fever (70).

Rarely, the muscular dystrophies caused by *DMD* mutations (71, 72), limb girdle muscular dystrophy due to *FKRP, ANO5, CPN3* (73)*, CAV3* (74), *DYSF* (75) and sarcoglycanopathies (76), and channelopathies secondary to *RYR1* (77, 78) mutations, can present in a “pseudometabolic fashion” (Figure 3) (60). Dystrophin, encoded by the *DMD* gene, is a component of the dystrophin-associated glycoprotein complex which accumulates at the NMJ and synapses and has a structural function in stabilizing the sarcolemma and synaptic transmission. Patients carrying *DMD* mutations and exhibiting this particular phenotype presented with or without fixed muscle weakness or calf hypertrophy and frequently carried exon deletions throughout the gene or particularly mutations involving the proximal third of the rod domain (71, 72). Rhabdomyolysis triggered by exercise, heat, or infections and a mild proximal weakness and ptosis is detected in some patients carrying heterozygous *RYR1*mutations, encoding for the principal sarcoplasmic reticulum calcium release channel, with a fundamental role in excitation-contraction coupling. *RYR1* mutations are a common cause of neuromuscular disease, ranging from various congenital myopathies to the malignant hyperthermia (MH) susceptibility. It is therefore important to these identify patients (77, 78). Finally, specific polymorphic variants in a number of genes have been reported in association with increased susceptibility to exertional rhabdomyolysis, together with exceptional athletic abilities and including *ACE, ACTN3, CCL2* and *CCR2* genes (35).

#### Myasthenia Gravis and Congenital Myasthenic Syndromes

An abnormal fatigability or fluctuating muscle weakness that worsens after exertion suggests a suspect of myasthenia gravis (MG) or congenital myasthenic syndromes (CMS) (Figure 3). They are respectively autoimmune and genetic diseases caused by impairment of synaptic transmission in the NMJ (79).

MG is an autoimmune disease caused by antibodies directed against key molecules at the NMJ, such as the nicotinic acetylcholine receptor (AChR), muscle-specific kinase (MuSK), and low-density lipoprotein receptor-related protein 4 (Lrp4), or agrin in the postsynaptic membrane. Those autoantibodies alter densities or function of AChRs and neuromuscular transmission. Consequently, they result in a localized or generalized fluctuating weakness that primarily affects ocular muscle with ptosis and diplopia (80). Fatigability can be elicited during examination by prolonged upgaze or repetitive movements; improvement of ptosis after the application of ice in a latex glove to the eyes can help in distinguishing myasthenia from other causes of ptosis (79). if suspected in a clinical setting, they are confirmed by positive antibody tests and electrophysiological tests, including single-fiber electromyography (SFEMG), and repetitive nerve stimulation (RNS), but routine nerve conduction tests and EMG are not usually informative. Good prognosis is obtained with adequate and prompt symptomatic treatment with AChE inhibitor (pyridostigmine), thymectomy and immunomodulatory treatments including corticosteroids, immunoglobulin (IVIg), plasma exchange and immunosuppressants being mycophenolate mofetil and azathioprine the most commonly used (80–82). Although onset ranges from birth to adulthood, MG during childhood is very infrequent, and the diagnosis is challenging because of the higher percentage of seronegative patients and the possible differential diagnosis of CMS (81).

CMS comprise a group of rare early onset hereditary disorders caused by mutations in several genes that code for constitutive proteins of the NMJ and are then characterized by impaired neuromuscular transmission (83, 84). The phenotype is typically dominated by fatigable muscle weakness, frequently combined with ptosis, ophthalmoparesis, facial, bulbar or generalized muscle weakness, spinal deformities and reduced muscle bulk; cognitive disability, dysmorphism, neuropathy, or epilepsy are occasional (85). The diagnosis is secured by SFEMG or RNS demonstrating a neuromuscular transmission defect (Figure 3). Mutation in more than 30 causative genes has been identified, mostly harbored in an autosomal recessive pattern (84). CMS are classified according to the location of the mutated protein as presynaptic, synaptic or postsynaptic ant, but new gene coding for ubiquitous proteins have been identified recently, including those involved in the glycosylation pathways and the synthesis of propyloligopeptidase (84). The most common causative gene is *CHRNE*, which accounts for 30–50% of the CMS cases, followed by *RAPSN, COLQ, DOK7, CHAT*, and *GFPT1* (85). Genotype-phenotype correlations are difficult. However, some peculiar phenotypic aspects may point toward a specific genetic defect, such as episodic apneas (*CHAT, RAPSN*), tongue atrophy and stridor and vocal cord paralysis in neonates or infants (*DOK7*), congenital contractures (*RAPSN, AChR* δ or γ subunit, *CHAT*), limb-girdle and axial distribution of weakness (*DOK7, GFPT1, DPAGT1, ALG2, ALG14*), facial dysmorphism (*SYB1, RAPSN, SCN4A, COLQ)*, seizures or ID (*DPAGT1*). Interestingly, a mutation in *SCN4A*, encoding for a post-synaptic sodium channel responsible for the generation of membrane action potentials, have been linked to CMS but also to hyperkalaemic or hypokalaemic periodic paralysis, myotonia presentation and congenital myopathy or fetal akinesia (86). The main symptomatic treatment in most CMS subtypes, such as AChR deficiency, fast-channel syndrome and rapsyn deficiency is the AChE inhibitor (pyridostigmine), which prolongs synaptic response to Ach. It is, however, ineffective or dangerous in CMS due to mutations in *COLQ, DOK7* and *CHRNA1*. 3,4-Diaminopyridine, a potassium channel blocker that enables the release of ACh from the presynaptic terminal, is used as an adjunctive treatment with pyridostigmine and ChAT CMS could theoretically worsen in response. Adrenergic agonists are effective in DOK7 and AChE deficiency through stabilization of the postsynaptic membrane. Long-lived open-channel blockers of the AChR ion channel (fluoxetine and quinidine) are used in the slow-channel syndrome (83). Therefore, genetic characterization of CMS is central for selecting the adequate tailored treatment (83–85).

#### Primary Muscle Channellopathies

Primary muscles channelopathies are a group of inherited disorders caused by mutations in genes encoding sodium channel (*SCN4A*), chloride channel (*CLCN1*), calcium channel (*CACNA1S*), or potassium channel (*KCNJ2* and *KCNJ18*) (87). They comprise two major categories: non-dystrophic myotonia (NDMs) and periodic paralysis (PP) (88). Despite different pathophysiology, the cardinal clinical manifestations of these disorders are muscle weakness or stiffness elicited or worsened by exercise.

Myotonia, the cardinal features of NDMs, is defined by an involuntary muscle contraction that persists for several seconds after cessation of voluntary effort and causes stiffness, impaired mobility and rarely pain. Myotonia can involve the eye, facial and jaw muscles as well as the arms and legs. Myotonic stiffness decreased with continued voluntary muscle activity (warm-up phenomenon), but sometimes myotonic stiffness may paradoxically worsen by repeated muscle activity (paramyotonia).

The NDMs include myotonia congenita (MC) due to mutations in the skeletal muscle chloride channel gene *CLCN1*, paramyotonia congenita (PMC) and sodium channel myotonia (SCM), both of the latter are caused by mutations in the skeletal muscle sodium channel gene *SCN4A* encoding Nav1.4. Chloride channel myotonia can be inherited in a recessive or dominant pattern (Becker's and Thomsen's myotonia, respectively), while myotonia due to sodium channel mutations is always autosomal dominant (89). Interestingly, exercise improves symptoms in MC, while it worse them in *SCN4A*-related disorders. In individuals with Thomsen disease, myotonia, associated muscle rigidity, and muscle hypertrophy may become apparent from infancy to approximately 2–3 years of age, while in those with Becker disease, age of onset is usually later (89). In severe myotonic disorders, patients develop a body-builder-like appearance secondary to continuous myotonic contractions.

The absence of severe fixed weakness or muscle wasting distinguishes NDMs from dystrophic myotonias, such as myotonic dystrophies type 1 and type 2 (DM1 and DM2 due to *DMPK* and C*N*BP mutations, respectively), which present with both progressive muscle weakness and multisystem involvement (Figure 4) (87). Schwartz-Jampel Syndrome should be considered in the differential diagnosis in patients with severe myotonia not associated with the warm-up phenomenon and dysmorphic features, such as short stature, muscular hypertrophy, joint contractures, bone dysplasia, ocular and facial abnormalities. This condition is caused by a loss-of-function mutation in the *HSPG2* gene, which encodes perlecan, a major component of basement membranes (90).

PMC is characterized by episodes of sustained myotonia that, unlike other forms, worsen by exercise and repeated movements and exposure to cold temperatures. Stiffness most commonly affects the muscles in the neck, face, arms and hands. Intermittent periods of muscle weakness (flaccid paresis) have also been reported (89).

The intensity and the duration of the myotonic stiffness are variable and may also depend upon other trigger factors such as temperature, emotional stress, and concomitant medical conditions. Especially in children, myotonia causing rigidity and stiffness of a body segment after a voluntary movement can be difficult to distinguish from paroxysmal dyskinesia (91). The first patient reported in the literature with paroxysmal dyskinesias was defined as having myotonia congenita (92). Treatment of myotonia includes pharmacological agents (mexiletine at the first line, but also lamotrigine, carbamazepine and acetazolamide) as well as dietary and lifestyle precautions (93).

The PPs are a group of skeletal muscle channelopathies characterized by intermittent attacks of muscle weakness often associated with altered serum potassium levels (Figure 4). Primary periodic paralysis (PP) include hypokalemic paralysis (HypoKPP), hyperkalemic paralysis (HyperKPP), and Andersen-Tawil syndrome (ATS) (94). They are caused by mutations in the skeletal muscle sodium, calcium, and potassium channels and have an autosomal dominant inheritance. Despite genetic heterogeneity, all forms share a common final mechanistic pathway, aberrant depolarization and muscle fiber unexcitability (89). Typically, attacks of paralysis are provoked by exercise or periods of rest (minutes to hours) after vigorous exercise but also infection, stress, fatigue, menses can trigger the attacks. Onset is during the first or second decade and the frequency of episodes is variable but tends to reduce over time. Between episodes patients are neurologically normal until adulthood, when they may develop progressive proximal fixed weakness.

HypoKPP, the most common form of PPs, is caused by mutations in the alpha subunits of either the skeletal muscle L-type calcium channel gene *CACN1AS* (HypoKPP1) or the skeletal muscle sodium channel gene *SCN4A* (HypoKPP2) (94). Patients with HypoKPP tend to have prolonged and severe attacks of weakness (several hours to days or weeks), focal or generalized, usually sparing facial and respiratory muscles. Symptom onset is typically in the second decade. Besides exercise, carbohydrate-rich meals are a typical trigger (95). During episodes, potassium levels are constantly reduced (<3.0 mM) and potassium supplements are part of the treatment regime, while the reverse is true for HyperPP where serum potassium levels are usually elevated and attacks can be triggered by potassium and ameliorated by glucose.

Patients with HyperKPP exhibit an earlier onset. Attacks are characterized by generalized weakness lasting hours, sometimes with fatal involvement of bulbar muscle. Electrical and clinical myotonia is a useful diagnostic pointer as it is often associated with HyperKPP and not found in HypoKPP. HyperKPP is caused by mutations in *SCN4A*, as well as the allelic disorder paramyotonia congenital.

ATS is characterized by the clinical triad of episodic flaccid muscle weakness, ventricular arrhythmias and prolonged QT interval and dysmorphic features. These include short stature, scoliosis, low-set ears, hypertelorism, broad nasal bridge, micrognathia, clino- or syndactyly, and toes joined at the base. Myotonia is not present. Patients may exhibit either hypokaliemic or hyperkaliemic paralysis. Around 60% of ATS patients carried a mutation in the potassium inward rectifier *KCNJ2* gene on chromosome 17q24 (87, 95).

### Epilepsy

Although epilepsy is characterized by recurrent, unprovoked seizures, a number of endogenous or exogenous precipitant factors have been identified. They include sleep deprivation, intercurrent illnesses, menstrual cycle, alcohol intake, protracted fasting and photic stimulation. Physical exhaustion due to exercise or sports activity has also been considered. Its causative role has primarily been attributed to hyperventilation, hypoxia, hypercapnia, hyperthermia, hypoglicemia and hyponatremia (96, 97). There are very few reports of adult patients with clear exercise-induced seizures, and interestingly most of them demonstrated temporal lobe partial epilepsy (98, 99). Report of pediatric cases are even more scarce (100), but the possibility that a paroxysmal event precipitated by exercise may be epileptic in nature has to be taken into account in the differential diagnosis of PENS.

### Psychogenic Disorders

The term psychogenetic or functional disorders encompass symptoms and disturbances of motor function that are not explained by organic conditions (101).

Psychogenetic disorders in adult neurological patients are well-described and characterized in the literature, but in recent years functional phenomenology in pediatric neurology has emerged as an important topic frequently encountered in clinical practice (101).

Psychogenic paroxysmal movement disorders are characterized by episodes that usually have multiple or inconsistent triggers in respect of primary paroxysmal dyskinesias; onset of the clinical symptoms is often acute following a stressor life event. The duration of episodes is also variable and patients often present with other unexplained somatic symptoms (102).

Common functional symptoms are also weakness, fatigue, paralysis, myalgias and cramps, which may lead clinicians to initially suspect a possible neuromuscular disorder (56). The diagnosis of a functional neuromuscular disorder is based upon the inconsistency of the clinical symptoms and the neurological examination, with the support, whenever appropriate, of medical tests.

## Discussion

A wide range of neurological symptoms often encountered in clinical practice are typically elicited by physical exercise. Unfortunately, being paroxysmal and not easily observed during an examination, the characterization of PENS is based on a clinical description from parents and patients; however, the partial resemblance among symptoms and the extensive genetic heterogeneity associated make the differential diagnosis frequently difficult and delayed. However, reaching a diagnosis is mandatory due to the availability of specific treatment. We suggest practical algorithms which could help orient the diagnosis.

The first step consists of correctly recognizing the clinical phenomenology and classifying it within three main categories of PENS: paroxysmal moment disorder, exercise intolerance or a paroxysmal exercise induced stiffness /weakness; and we propose a flowchart for each clinical entity (**Figures 2–4**).

An accurate assessment of the age at onset, type and duration of the triggering exercise, the presence of non-paroxysmal neurological symptoms and/or multisystemic involvement are fundamental for guide a multi-step path that requires the support of neuroradiologic, electrophysiological and laboratory investigations.

While in adolescents a PARK2 should be investigated through a DATscan, in childhood Brain MRI is fundamental to exclude symptomatic PED and investigate specific abnormalities in basal ganglia that can direct genetic testing for neurometabolic disorders. In patients with normal brain MRI, a lumbar puncture would be crucial for exploring DRD and GLUT1 syndrome (Figure 2). In patients with exercise intolerance particular clinical key signs or symptoms, such as myoglobinuria, “second wind phenomenon,” the presence of baseline neurological or multisystemic involvement and the type of duration of exercise inducing those symptoms are the most relevant information for address diagnostic suspicion of metabolic myopathies rather than other neuromuscular disorders, including myasthenia and muscular dystrophies (Figure 3). In most cases, histological and immunohistochemical analysis on muscle biopsy is fundamental to addressing specific genetic testing. Finally, the clinical and/or electrophysiological evidence of myotonia or paramyotonia, together with the detection of other muscular or extramuscular features and altered serum potassium levels could expedite the diagnostic process (Figure 4). In some cases, such as EAs or Myotonic Dystrophies, the characterization of the specific motor phenomenon in the context of a positive familial history could directly suggest specific genetic confirmation. If a defined etiological diagnosis cannot be reached after extensive work-up, especially when the paroxysmal phenomenon does not fit properly into a precise category, the possibility of a psychogenic disorder has to be ruled out.

Intriguingly, despite the wide clinical and genetic heterogeneity described herein, it is possible to identify some common pathogenic mechanisms responsible for these episodic neurological disorders. The defects in energy metabolism that impair the ability to respond to increased energy needs in the brain and muscles during exercise appear to be crucial pathogenic mechanisms for PED due to *SLC2A1* mutations and mitochondrial disorders (including defect of PDH complex, ECSH1 and HIBCH) and metabolic myopathies. Secondly, a number of PENS grouped as “channelopathies,” due to mutations in either central and muscular ion channels or their interacting proteins (103, 104). These mutations result in the alteration of action potential properties or synaptic transmission; several stressors, including exercise, can prompt an unstable and symptomatic state. Consistent with the distribution of ion channels throughout the human body and their resulting hyper- or hypoexcitability, channelopathies manifest with a variety of PENS, including movement disorders and myotonia, myasthenia and periodic paralysis (86, 104). Moreover, mutations in several PENS-associated genes such as *PRRT2, MR-1, ADCY5* and *TBC1D24* results in alterations of the synaptic function and regulation of vesicle trafficking and neurotransmitter release (105). Finally, impaired neurotransmission due to the defect of dopamine and GABA metabolism (*PARKIN, GCH and ALDH5A1* mutations) is responsible for exercise- induced movement disorders.

Finally, we propose a new clinical entity named PENS and reviewed clinical features, genetic and principles of treatment mainly focusing on phenomenology and clues for differential diagnosis. Given the overlapping clinical features and molecular bases, considering PENS as a whole would help consider the entire range of differential diagnosis.

## Author Contributions

GZ, FD, NN, and BG contributed to conception and design of the study. GZ and FD wrote the first draft of the manuscript. IM and FI wrote sections of the manuscript. All authors contributed to manuscript revision, read, and approved the submitted version.

## Conflict of Interest

The authors declare that the research was conducted in the absence of any commercial or financial relationships that could be construed as a potential conflict of interest.
